# Association of sleep duration in pregnancy with preterm birth in China: a cross-sectional survey

**DOI:** 10.3389/fped.2025.1493248

**Published:** 2025-06-09

**Authors:** Hui Jiang, Bin Yu, Yuanyang Liu, Hui Gao, Ruijuan Song, Siyue Tan, Shufen Han, Hui Zuo

**Affiliations:** ^1^ School of Public Health, Suzhou Medical College of Soochow University, Suzhou, China; ^2^ Medical Research Center, Sichuan Bingzhe Technology Co., Ltd., Chengdu, China; ^3^ School of Public Health, Hangzhou Normal University, Hangzhou, Zhejiang, China; ^4^ Jiangsu Key Laboratory of Preventive and Translational Medicine for Major Chronic Non-communicable Diseases, Suzhou Medical College of Soochow University, Suzhou, China; ^5^ MOE Key Laboratory of Geriatric Diseases and Immunology, Suzhou Medical College of Soochow University, Suzhou, China

**Keywords:** sleep duration, preterm birth, pregnant women, cross-sectional survey, China

## Abstract

**Introduction:**

Only a few studies have reported the relationship between the sleep duration of pregnant women and preterm birth (PTB), and the findings are inconsistent. This study aimed to examine the association of maternal sleep duration in pregnancy with PTB in China.

**Methods:**

A cross-sectional survey. Sleep duration in pregnancy and PTB status were self-reported via a validated questionnaire. The association of sleep duration in pregnancy with PTB was examined by logistic regression models.

**Results:**

The overall prevalence of PTB was 16.6%. Compared to the women with total sleep duration in pregnancy of >8 h/day, those with sleep duration of 7–8 h/day (OR = 1.43; 95% CI: 1.20, 1.70) and <7 h/day (OR = 4.28; 95% CI: 3.06, 6.00) had higher odds of reporting PTB after adjustment for gender of baby, maternal age at delivery, educational level of mother, educational level of father, diabetes history, gestation diseases including gestational hyperglycemia, gestational hypertension, anemia during pregnancy and anxiety or depression during pregnancy, and pregnancy behaviors including smoking or passive smoking during pregnancy, alcohol drinking during pregnancy, prenatal education, exercise during pregnancy and folic acid supplementation daily during pregnancy. The risk associations were similar in subgroups stratified by sex, gestational hyperglycemia, anemia during pregnancy and status of folic acid supplementation.

**Discussion:**

Sleep duration in pregnancy was inversely associated with PTB in China. Our novel findings suggest the importance of sufficient sleep in pregnant women for the prevention of PTB. Further studies are warranted to confirm and elucidate the observed association.

## 1 Introduction

Preterm birth (PTB) is defined by the World Health Organization as births before 37 completed weeks of gestation or less than 259 days from the first date of a woman's last menstrual period (1). It is one of the leading causes of newborn death (2, 3). Additionally, premature infants who survive have higher rates of long-term morbidity, including neurologic and developmental disabilities (4). In the United States, the prevalence of cerebral palsy among preterm infants is about 2‰, and the prevalence is as high as 3.4‰ in other regions, which can bring an enormous burden to patients, families and society (5, 6). The PTB rate has become an important indicator of perinatal health in a country or region. Overall, the prevalence of PTB in the world is on the rise, and the global PTB rate increased from 9.8% in 2000 to 10.6% in 2014 (7, 8). It is reported that China has the second highest number of preterm birth in the world, with more than one million premature infants each year (9).

Previous studies have shown multiple factors associated with PTB, including maternal demographic characteristics, nutritional status, psychological characteristics, adverse behaviors, infection, and biological markers (10). However, there are only a few studies on the relationship between sleep duration in pregnant women and PTB, and the existing results are inconsistent (11, 12). For example, as shown in a research, participants with short duration of sleep during the third trimester were more likely to report preterm birth (13). In contrast, other researches showed that the relationship between long/short sleep duration and preterm birth had no significant (14, 15). Thus, we aimed to examine the association of sleep duration during pregnancy with PTB among Chinese women, provide more evidence for the association between sleep duration and preterm birth.

## 2 Materials and methods

### 2.1 Study participants

The present study was based on a cross-sectional survey in October-December 2021. A total of 30 kindergartens in Chengdu, a provincial capital in Southwest China, were selected by a cluster random sampling method. Data were collected by a validated questionnaire administered by the mothers of preschool children via WJX (an online survey platform, https://www.wjx.cn). Overall, 4,360 mothers participated in the survey and were finally included in the analysis.

The study protocol was approved by the Ethics Committee of Soochow University (Approval NO. SUDA20210820H01). The questionnaire opens with the statement “answering the questionnaire constitutes consent to participate” in the study.

### 2.2 Assessment of sleep duration in pregnancy

Information on sleep duration in pregnancy was obtained via the following question: “For how long did you sleep including siesta per day during pregnancy? (a) <7 h/day, (b) 7–8 h/day, (c) >8 h/day” (16, 17).

### 2.3 Definition of PTB

The PTB status was determined by the following question in the questionnaire: “How many gestational weeks did you have when you delivered your child? (a) ≥37 weeks, (b) <37 weeks”. Gestational weeks of <37 weeks were defined as PTB (1).

### 2.4 Covariates

Gender of baby (male and female), maternal age at delivery (<25, 25–29, 30–34, and ≥35 years), educational level of mother (lower secondary education or below, high school or secondary vocational education, associate degree, bachelor's degree or above), educational level of father (lower secondary education or below, high school or secondary vocational education, associate degree, bachelor's degree or above), diabetes history (yes and no), gestation diseases including gestational hyperglycemia (yes and no), gestational hypertension (yes and no), anemia during pregnancy (yes and no) and anxiety or depression during pregnancy (yes and no), and pregnancy behaviors including smoking or passive smoking during pregnancy (0, 1–2, 3–4, and ≥5 days/week), alcohol drinking during pregnancy (0, 1–2, 3–4, and ≥5 days/week), prenatal education (0, 1–2, 3–4, and ≥5 times/week), exercise during pregnancy (0, <20, 20–40, and >40 min/day), and folic acid supplementation daily during pregnancy (yes and no) were all collected by self-report via the questionnaire (18, 19).

### 2.5 Statistical analyses

The basic characteristics of the participants by PTB status and sleep duration in pregnancy are presented as percentages (%) and were compared using the chi-square test or Fisher's exact test.

We used logistic regression to evaluate the association of sleep duration in pregnancy with PTB. Three models were constructed: model 1 was the basic model adjusted for sex of the baby, maternal age at delivery, educational level of mother and educational level of father; model 2 was adjusted as model 1 plus diabetes history and gestation diseases, including gestational hyperglycemia, gestational hypertension, anemia during pregnancy and anxiety or depression during pregnancy; and model 3 was adjusted as model 2 plus pregnancy behaviors, including smoking or passive smoking during pregnancy, alcohol drinking during pregnancy, prenatal education, exercise during pregnancy and folic acid supplementation daily during pregnancy. The *P*-trend was calculated by modelling the independent variable as a continuous variable in the regression models.

Effect modification of the association between sleep duration in pregnancy and PTB by gender of baby, gestational hyperglycemia, anemia during pregnancy and folic acid supplementation daily during pregnancy was examined by stratified analyses, and significance of interactions was evaluated on the first-degree multiplicative models for each stratification variable separately.

The statistical analyses were performed using SPSS 26.0 statistical software (IBM Corp., USA). All tests were two-tailed, and a *P* value <0.05 was considered to indicate statistical significance.

## 3 Results

Of the 4,360 women, there were 725 (16.6%) premature babies. The proportions of participants whose total sleep duration in pregnancy was <7, 7–8, and >8 h/day were 3.8%, 33.6%, and 62.6%, respectively.

Table 1 shows the characteristics of the participating women by PTB status. The likelihood of PTB was highest in women whose delivery age was ≥35 years old (*P* = 0.039). The prevalence of preterm birth was significantly higher for infants whose mother's or father's educational attainment was lower secondary education or below (*P* < 0.001). Pregnant women with gestational hypertension were more likely to have PTB (*P* = 0.004). Moreover, the prevalence of PTB was higher in women with less prenatal education (*P* = 0.023) and exercise during pregnancy (*P* < 0.001).

**Table 1:** Characteristics of the participants according to PTB status.

Characteristics	Overall	Non-PTB	PTB	*P* value
Participants, *N*	4,360 (100)	3,635 (83.4)	725 (16.6)	
Sex (%)				
Male	2,274 (52.2)	1,890 (52.0)	384 (53.0)	0.63
Female	2,086 (47.8)	1,745 (48.0)	341 (47.0)	
Maternal age at delivery (years)				
<25	1,042 (23.9)	853 (23.5)	189 (26.1)	0.039
25–29	2,079 (47.7)	1,767 (48.6)	312 (43.0)	
30–34	860 (19.7)	710 (19.5)	150 (20.7)	
≥35	379 (8.7)	305 (8.4)	74 (10.2)	
Educational level of mother (%)				
Lower secondary education or below	594 (13.6)	439 (12.1)	155 (21.4)	<0.001
High school or secondary vocational education	1,436 (32.9)	1,152 (31.7)	284 (39.2)	
Associate degree	1,262 (28.9)	1,098 (30.2)	164 (22.6)	
Bachelor's degree or above	1,068 (24.5)	946 (26.0)	122 (16.8)	
Educational level of father (%)				
Lower secondary education or below	607 (13.9)	468 (12.9)	139 (19.2)	<0.001
High school or secondary vocational education	1,405 (32.2)	1,151 (31.7)	254 (35.0)	
Associate degree	1,131 (25.9)	960 (26.4)	171 (23.6)	
Bachelor's degree or above	1,217 (27.9)	1,056 (29.1)	161 (22.2)	
Diabetes history (%)				
Yes	86 (2.0)	66 (1.8)	20 (2.8)	0.10
No	4,274 (98.0)	3,569 (98.2)	705 (97.2)	
Gestational hyperglycemia (%)				
Yes	598 (13.7)	486 (13.4)	112 (15.4)	0.14
No	3,762 (86.3)	3,149 (86.6)	613 (84.6)	
Gestational hypertension (%)				
Yes	156 (3.6)	117 (3.2)	39 (5.4)	0.004
No	4,204 (96.4)	3,518 (96.8)	686 (94.6)	
Anemia during pregnancy (%)				
Yes	1,053 (24.2)	883 (24.3)	170 (23.4)	0.63
No	3,307 (75.8)	2,752 (75.7)	555 (76.6)	
Anxiety or depression during pregnancy (%)				
Yes	115 (2.6)	92 (2.5)	23 (3.2)	0.33
No	4,245 (97.4)	3,543 (97.5)	702 (96.8)	
Smoking or passive smoking during pregnancy (days/week)				
0	4,072 (93.4)	3,387 (93.2)	163 (94.5)	0.33
1–2	185 (4.2)	163 (4.5)	22 (3.0)	
3–4	46 (1.1)	39 (1.1)	7 (1.0)	
≥5	57 (1.3)	46 (1.2)	11 (1.5)	
Alcohol consumption during pregnancy (days/week)				
0	4,333 (99.4)	3,615 (99.4)	718 (99.0)	0.10
1–2	11 (0.3)	8 (0.2)	3 (0.4)	
3–4	10 (0.2)	9 (0.2)	1 (0.2)	
≥5	6 (0.1)	3 (0.2)	3 (0.4)	
Prenatal education (times/week)				
0	575 (13.2)	462 (1 2.7)	113 (15.6)	0.023
1–2	1,933 (44.3)	1,601 (44.0)	332 (45.8)	
3–4	1,159 (26.6)	972 (26.7)	187 (25.8)	
≥5	693 (15.9)	600 (16.6)	93 (12.8)	
Exercise during pregnancy (minutes/day)				
0	298 (6.8)	227 (6.2)	71 (9.8)	<0.001
<20	670 (15.4)	541 (14.9)	129 (17.8)	
20–40	1,832 (42.0)	1,512 (41.6)	320 (44.1)	
>40	1,560 (35.8)	1,355 (37.3)	205 (28.3)	
Folic acid supplementation daily during pregnancy (%)				
Yes	3,860 (88.5)	3,228 (88.8)	632 (87.2)	0.21
No	500 (11.5)	407 (11.2)	93 (12.8)	
Sleep duration in pregnancy (hours/day)				
<7	167 (3.8)	93 (2.6)	74 (10.2)	<0.001
7–8	1,466 (33.6)	1,191 (32.8)	275 (37.9)	
>8	2,727 (62.6)	2,351 (64.6)	376 (51.9)	

The variables are expressed as numbers (*n*) and percentages (%). *P* was determined by Chi-square test or Fisher's exact test. PTB, preterm birth.

The characteristics of the participants in terms of sleep duration in pregnancy are presented in Table 2. Maternal age at delivery (*P* < 0.001), educational level of mother (*P* < 0.001), educational level of father (*P* < 0.001), gestational hypertension (*P* = 0.007), anxiety or depression during pregnancy (*P* = 0.001), smoking or passive smoking during pregnancy (*P* = 0.047), alcohol consumption during pregnancy (*P* = 0.002), prenatal education (*P* < 0.001), and exercise during pregnancy (*P* < 0.001) were different between the groups.

**Table 2:** Characteristics of the participants according to sleep duration in pregnancy.

Characteristics	Sleep duration in pregnancy (h/day)			*P* value
	<7	7–8	>8	
Participants, *N*	167 (3.8)	1,466 (33.6)	2,727 (62.6)	
Sex (%)				
Male	76 (45.5)	767 (52.3)	1,431 (52.5)	0.21
Female	91 (54.5)	699 (47.7)	1,296 (47.5)	
Maternal age at delivery (years)				
<25	43 (25.7)	292 (19.9)	707 (25.9)	<0.001
25–29	66 (39.5)	723 (49.3)	1,290 (47.3)	
30–34	38 (22.8)	318 (21.7)	504 (18.5)	
≥35	20 (12.0)	133 (9.1)	226 (8.3)	
Educational level of mother (%)				
Lower secondary education or below	40 (6.7)	209 (35.2)	345 (58.1)	<0.001
High school or secondary vocational education	62 (4.3)	430 (29.9)	944 (65.7)	
Associate degree	36 (2.9)	387 (30.7)	839 (66.5)	
Bachelor's degree or above	29 (2.7)	440 (41.2)	599 (56.1)	
Educational level of father (%)				
Lower secondary education or below	34 (5.6)	214 (35.3)	359 (59.1)	<0.001
High school or secondary vocational education	55 (3.9)	439 (31.2)	911 (64.8)	
Associate degree	41 (3.6)	356 (31.5)	734 (64.9)	
Bachelor's degree or above	37 (3.0)	457 (37.6)	723 (59.4)	
Diabetes history (%)				
Yes	7 (4.2)	25 (1.7)	54 (2.0)	0.12
No	160 (95.8)	1,441 (98.3)	2,673 (98.0)	
Gestational hyperglycemia (%)				
Yes	27 (16.2)	208 (14.2)	363 (13.3)	0.47
No	140 (83.8)	1,258 (85.8)	2,364 (86.7)	
Gestational hypertension (%)				
Yes	13 (7.8)	44 (3.0)	99 (3.6)	0.007
No	154 (92.2)	1,422 (97.0)	2,628 (96.4)	
Anemia during pregnancy (%)				
Yes	49 (29.3)	337 (23.0)	667 (24.5)	0.16
No	118 (70.7)	1,129 (77.0)	2,060 (75.5)	
Anxiety or depression during pregnancy (%)				
Yes	13 (7.8)	35 (2.4)	67 (2.5)	0.001
No	154 (92.2)	1,431 (97.6)	2,660 (97.5)	
Smoking or passive smoking during pregnancy (days/week)				
0	153 (91.6)	1,376 (93.9)	2,543 (93.3)	0.047
1–2	5 (3.0)	57 (3.9)	123 (4.5)	
3–4	1 (0.6)	15 (1.0)	30 (1.1)	
≥5	8 (4.8)	18 (1.2)	31 (1.1)	
Alcohol consumption during pregnancy (days/week)				
0	162 (97.0)	1,457 (99.4)	2,714 (99.5)	0.002
1–2	2 (1.2)	2 (0.1)	7 (0.3)	
3–4	0 (0.0)	5 (0.3)	5 (0.2)	
≥5	3 (1.8)	2 (0.2)	1 (0.0)	
Prenatal education (times/week)				
0	42 (25.1)	221 (15.1)	312 (11.4)	<0.001
1–2	86 (51.5)	713 (48.6)	1,134 (41.6)	
3–4	30 (18.0)	374 (25.5)	755 (27.7)	
≥5	9 (5.4)	158 (10.8)	526 (19.3)	
Exercise during pregnancy (minutes/day)				
0	32 (19.2)	113 (7.7)	153 (5.6)	<0.001
<20	45 (26.9)	307 (20.9)	318 (11.7)	
20–40	62 (37.1)	664 (45.3)	1,106 (40.5)	
>40	28 (16.8)	382 (26.1)	1,150 (42.2)	
Folic acid supplementation daily during pregnancy (%)				
Yes	144 (86.2)	1,285 (87.7)	2,431 (89.1)	0.22
No	23 (13.8)	181 (12.3)	296 (10.9)	

The variables are expressed as numbers (*n*) and percentages (%). *P* was determined by Chi-square test or Fisher's exact test.

Compared with the women whose total sleep duration in pregnancy was >8 h/day, the multivariable adjusted ORs were 1.43 (95% CI: 1.20, 1.70) and 4.28 (95% CI: 3.06, 6.00) for those whose sleep duration in pregnancy was 7–8 and <7 h/day, respectively (*P*-trend <0.001) (Table 3).

**Table 3:** ORs (95% CI) for PTB by sleep duration in pregnancy.

	Cases/*N*	Model 1	Model 2	Model 3
Sleep duration in pregnancy (h/day)				
>8	376/2,727	1.00 (ref.)	1.00 (ref.)	1.00 (ref.)
7–8	275/1,466	1.50 (1.26, 1.78)	1.51 (1.27, 1.79)	1.43 (1.20, 1.70)
<7	74/167	4.68 (3.37, 6.50)	4.68 (3.37, 6.52)	4.28 (3.06, 6.00)
*P*-trend		<0.001	<0.001	<0.001

Model 1 was adjusted for sex, maternal age at delivery, educational level of mother and educational level of father. Model 2 was adjusted for model 1 plus diabetes history, gestational hyperglycemia, gestational hypertension, anemia during pregnancy and anxiety or depression during pregnancy. Model 3 was adjusted for model 2 plus smoking or passive smoking during pregnancy, alcohol consumption during pregnancy, prenatal education, exercise during pregnancy and folic acid supplementation daily during pregnancy. PTB, preterm birth.

**Figure 1:**
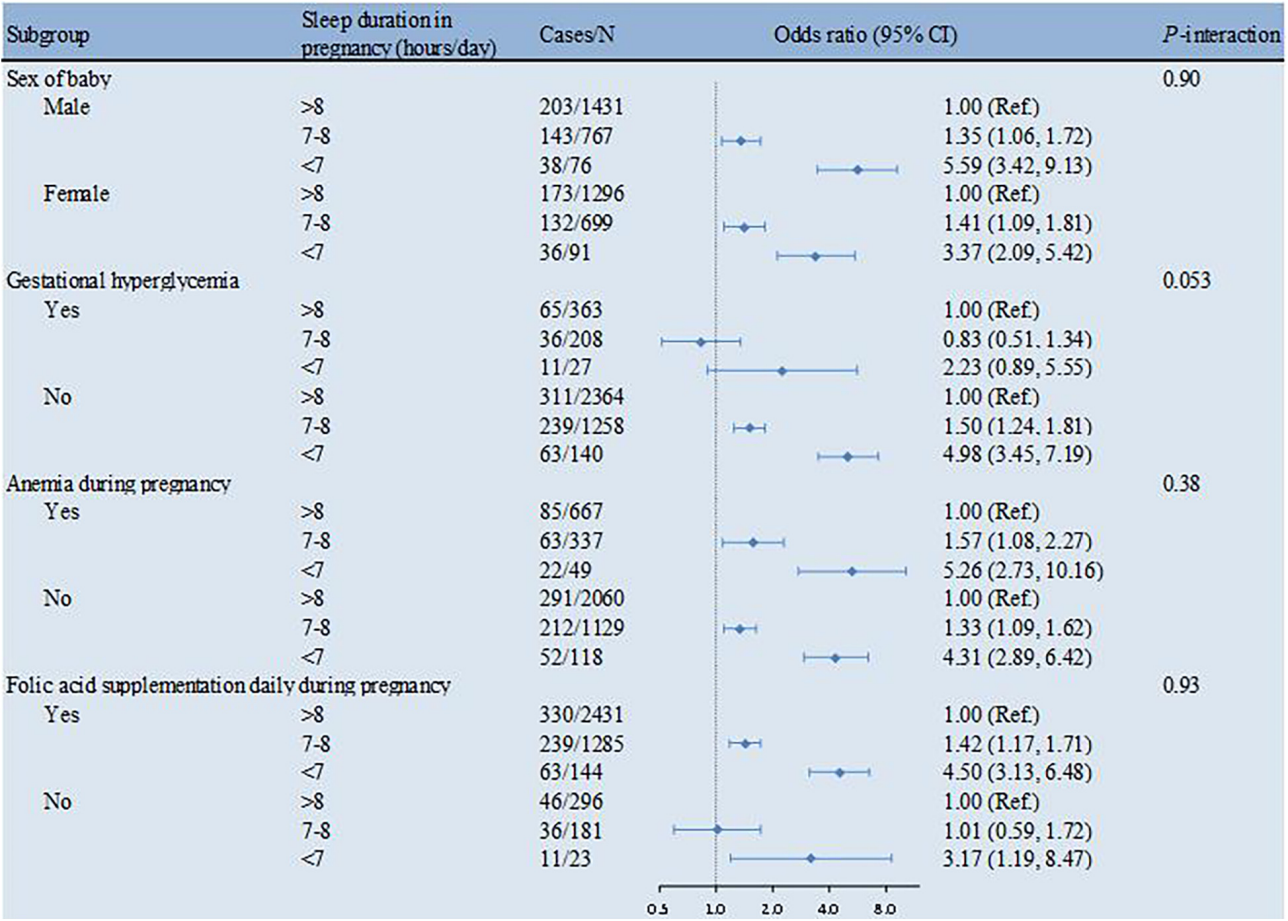
Sleep duration in pregnancy in association with PTB by strata. The multivariable model was adjusted for sex of the baby, maternal age at delivery, diabetes history, gestational hyperglycemia, gestational hypertension, anemia during pregnancy, anxiety or depression during pregnancy, smoking or passive smoking during pregnancy, alcohol consumption during pregnancy, prenatal education, exercise during pregnancy and folic acid supplementation daily during pregnancy. PTB, preterm birth.

The associations between sleep duration in pregnancy and PTB were similar in subgroups stratified by sex, gestational hyperglycemia, anemia during pregnancy and folic acid supplementation daily during pregnancy at baseline (all *P*-interaction >0.05) (Figure 1).

## 4 Discussion

### 4.1 Principal findings

In this cross-sectional study, we observed that short sleep duration in pregnancy was independently associated with increased odds of PTB among Chinese women. The association between sleep duration in pregnancy and PTB was independent of confounding factors and similar across subgroups stratified by gender of baby, gestational hyperglycemia, anemia during pregnancy and folic acid supplementation daily during pregnancy.

### 4.2 Sleep duration in pregnancy and PTB

Several studies have demonstrated that insufficient sleep is potentially associated with an increased risk of preterm birth among pregnant women, which is consistent with our conclusion. A study from India showed that sleep duration in full-term women was longer than that in preterm women (20). In a cohort study, compared to pregnant women with sleep duration >8 h/day in the nighttime, those with sleep duration ≤5 h/day were more inclined to give preterm birth (21). In a case‒control study based on pregnant women in the first 6 months of pregnancy, compared to women with sleep duration in pregnancy of 7–8 h/day, those with sleep duration 6 h/day were at higher risk of PTB (22). In two prospective studies and one meta-analysis, sleep duration of <7 h/day significantly increased the odds of PTB (11, 13, 23).

Other studies have shown that both insufficient and excessive sleep duration increases the risk of preterm birth. A dose-response meta-analysis demonstrated a strong association between extreme sleep duration during pregnancy and preterm birth, characterized by a U-shaped relationship (24). Meanwhile, a Mendelian randomized analysis revealed that pregnant women sleeping less than 5 h per night or more than 10 h per night exhibited a higher risk of preterm birth (25).

However, a study from the USA showed no associations between the sleep duration of pregnant women at night and PTB, whether in the first, second or third trimester (12). Generally, the current research on preterm birth and sleep duration is inconclusive, which is consistent with the findings of another review (26).

Different study designs, study populations and methods of data collection may explain inconsistent findings.

### 4.3 Potential mechanisms

The underlying mechanism of sleep duration in pregnancy with PTB remains unclear. Most scholars believe that the immune system plays a mediating role between them (12, 27–30). Sleep disorders, either poor sleep quality or short sleep duration, interfere with the normal immune process, elevated levels of proinflammatory cytokines and enhanced inflammatory responses (30).

The dynamic equilibrium between Th1 (cell-mediated immunity) and Th2 (humoral immunity) at the fetal-maternal interface plays a key role in women's successful pregnancy. Th1 cells produce proinflammatory cytokines such as interleukin-1, 2, 6, 8, 12 and tumor necrosis factor *α*. Th2 produced anti-inflammatory cytokines such as interleukin-4, 5, 10, and 13 (31–33). During normal pregnancy, Th2 activity was much stronger than Th1 activity, which had potential protective effects in the fetal-maternal relationship. For instance, the injection of IL-10, a Th2-type cytokine, prevents fetal wastage in mice prone to fetal resorption via conspicuous downregulatory effects on Th1-type cytokines (34). However, immune dysregulation by a progressive shift toward Th1 predominance may initiate and intensify the cascade of proinflammatory cytokine release, including IL-1, 2, 6, 8 and TNF-α (31), which induces the production of prostaglandins, an important inducer of uterine contraction (35, 36).

In addition, other physical and psychological conditions, such as gestational diabetes (37, 38), gestational hypertension (39, 40), obesity (41, 42), poor psychological status including depression (43, 44) and hyperhomocysteinaemia (45, 46), may also play a role in the association between sleep duration in pregnancy and PTB.

### 4.4 Strengths and limitations

The main strengths of our study include a relatively large sample size and consideration of adjustment for a number of potential confounders. Meanwhile, the findings of the existing research are inconsistent. Our study provides additional reference evidence for research in this field and is meaningful. Sleep duration was collected by subjective estimation of the participants instead of objective measurement, which may lead to self-reporting bias. The gestational status in our study was retrospectively estimated; thus, recall bias might have occurred. Quality of sleep, such as sleep-disordered breathing, was not collected, which limited further exploration (47).

## 5 Conclusion

In this cross-sectional study, sleep duration in pregnancy was inversely associated with PTB among Chinese women. Our findings underline the importance of sufficient sleep (>8 h/day) for pregnant women in the prevention of PTB.

## Data Availability

The raw data supporting the conclusions of this article will be made available by the authors, without undue reservation.
